# Co-occurrence of bronchial papilloma and pulmonary sclerosing hemangioma in a male

**DOI:** 10.1186/s40064-016-3493-6

**Published:** 2016-10-19

**Authors:** Jin-mei Wang, Qing-hai You, Cheng-cheng Niu

**Affiliations:** 1 Anhui Medical University, 81 Meishang Road, Hefei, 230032 Anhui People’s Republic of China; 2 Department of Respiratory Medicine, The First Affiliated Hospital of Anhui Medical University, 218 Jixi Road, Hefei, 230022 Anhui People’s Republic of China; 3 Department of Nurse, The First Affiliated Hospital of Anhui Medical University, 218 Jixi Road, Hefei, 230022 Anhui People’s Republic of China

**Keywords:** Pulmonary sclerosing hemangioma, Bronchial papillomas, Benign tumor, Male

## Abstract

**Introduction:**

Bronchial papilloma and pulmonary sclerosing hemangioma (PSH) are rare tumors. The development of PSH combined with bronchial papilloma in lung is extremely rare. We herein presented a case of the co-occurrence of bronchial papilloma and PSH in a male.

**Case description:**

A 48-year-old man was referred to our department for further treatment of the productive purulent cough and fever. After bronchoscopy, the biopsy of the specimen showed a bronchial papilloma in the lumen of posterior segment of the right upper lobar bronchus. For computed tomography demonstrated a focal lesion with an air bubble in the posterior segment of the right upper lobe, a lobectomy was performed and PSH was diagnosed.

**Discussion:**

The report emphasizes the fact that even though some of bronchial papilloma and PSH may have a low prevalence, nonetheless, the low prevalence of both diseases in a male has meant that it has not been possible to explore the depth of association between them.

**Conclusions:**

The paper reports a case of PSH in a male suffering from bronchial papilloma which is the exceptional concurrence of these two extremely infrequent events.

## Background

Bronchial papilloma is a rare tumor that accounts for 0.38 % of all lung tumors and 7–8 % of all benign lung tumors. It is not uncommon to report a significant delay in making an appropriate diagnosis for the clinical presentation of this tumor is usually nonspecific. The clinical presentation ranges from cough, dyspnea at rest or with exertion, to stridor and upper airway obstruction (Yıldırım et al. 2015; Harris and Chalhoub 2011).

Pulmonary sclerosing hemangioma (PSH) is predominantly identified in middle-aged women and accounts for approximately 1 % of all benign pulmonary tumors (Goel et al. 2013). Most patients of PSH are asymptomatic with the lesion being found incidentally upon chest radiographs performed for alternative reasons. Additionally, only a few patients experience hemoptysis, dyspnea, cough or chest pain due to an enlargement of the tumor and compression of surrounding tissue (Guerra-Gutiérrez et al. 2007).

The development of PSH combined with other different tumors, such as bronchial papilloma in lung is extremely rare. In this paper, we describe the unique findings of our case in comparison with previously reported cases.

## Case presentation

**Fig. 1:**
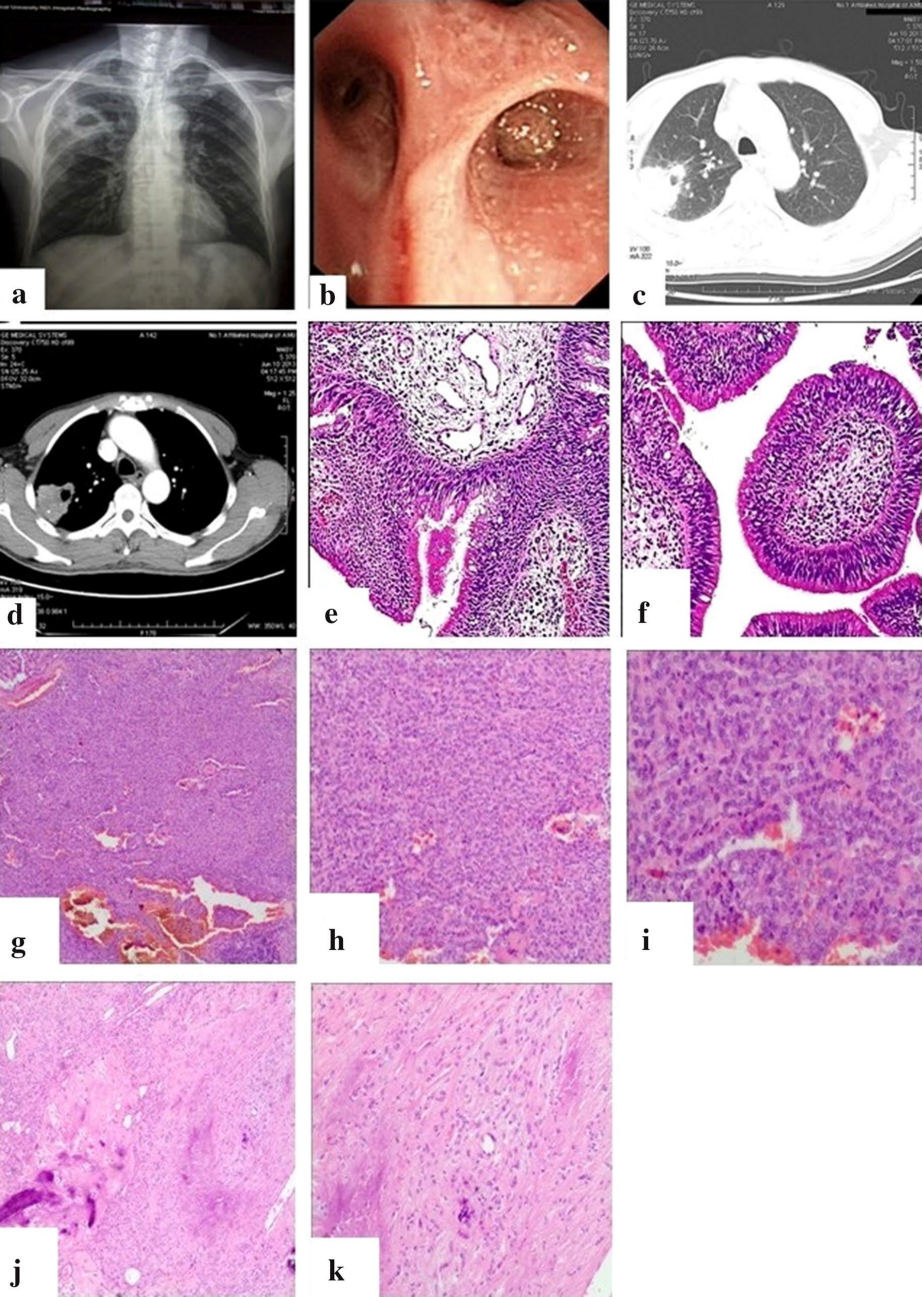
Imaging features and pathologic features of the patient. **a** Chest radiography shows focal round lesion with thick-walled cavity in the right upper lung. **b** Bronchoscopic picture of bronchial papilloma occluding the lumen of posterior segment of the right upper lobar bronchus. **c** Computed tomography shows a focal lesion of 4.0 × 4.4 cm in the posterior segment of the right upper lobe with broad-based contact to the pleura. **d** Computed tomography shows patchy calcification and marked contrast enhancement. Biopsy of the specimen shows bronchial papilloma in **e** (hematoxylin-eosin stain, ×100) and **f** (hematoxylin-eosin stain, ×40). Histopathological examination of the right upper lobe diagnoses of pulmonary sclerosing hemangioma, this image shows the solid areas in **g** (hematoxylin-eosin stain, ×40), **h** (hematoxylin-eosin stain, ×100) and **i** (hematoxylin-eosin stain, ×200). Histopathological examination of the right upper lobe diagnoses of pulmonary sclerosing hemangioma as following. **j** (hematoxylin-eosin stain, ×40) and **k** (hematoxylin-eosin stain, ×100) show the sclerosing areas

A 48-year-old man visited a local hospital because of productive purulent cough for seven days and fever for three days. Aside from these, the patient’s medical history was completely uneventful. Chest radiography performed prior to admission showed a focal round lesion with thick-walled cavity in the right upper lung (Fig. 1a). Some laboratory examinations except the leukocyte count 13,340/mm^3^ (83.91 % neutrophils), the erythrocyte sedimentation rate (67 mm/h), the C-reactive protein (106 mg/L) and the procalcitonin (0.106 ng/ml) were normal. Cultures for bacteria, fungus, and acid-fast baclilli were negative from the sputum. Pulmonary function examination is normal including FVC (3.27 L), FEV_1_ (3.06 L), FEV_1_% FVC (93.64 %) and MVV (87.67 L/min). For chest radiography revealed features suggestive of lung abscess (Fig. 1a), laboratory examinations and the clinical symptoms also indicated infection, the patient was given intravenous levofloxacin and piperacillin/tazobactum for three days, and the temperature returned to normal, but productive purulent cough was still existed. Herein, the bronchoscopic examination was performed and revealed a red, glistening, nodular single lesion obstructed the lumen of posterior segment of the right upper lobar bronchus (Fig. 1b). Biopsy of the specimen showed bronchial papilloma (Fig. 1e, f). Then, computed tomography (CT) was performed and demonstrated a focal lesion of 4.0 cm × 4.4 cm in the posterior segment of the right upper lobe with broad-based contact to the pleura (Fig. 1c), an air bubble 15 mm in diameter connected to the bronchial system, and patchy calcification (Fig. 1d). For distal to the papilloma there was obstructive pneumonia with lung abscess and the definite treatment, surgical resection was selected and a right upper lobectomy was performed. Histopathological examination revealed a 4.5 cm × 3 cm × 2.3 cm sized mass and the diagnosis of PSH (Fig. 1g–k) was confirmed with positive expression of thyroid transcription factor-1 (TTF-1), epithelial membrane antigen (EMA), cytokeratin fragment (CK) 7, cytokeratin and CD31, negative expression of CD34, CK20, CK5/6, synaptophysin and chromogranin A. The patient had an uncomplicated postoperative course and remained asymptomatic 3 months afterwards.

## Conclusions

This case produces two interesting findings as following. Firstly, bronchial papilloma and PSH are rare tumors while the description of a new case enhances the understanding of these diseases. Secondly, relative to the previous literature, co-occurrence of bronchial papilloma and PSH in a middle aged male is rare.

It is usually presented as an endobronchial mass in the segmental bronchi when bronchial papilloma occurs in the tracheobronchial tree, which has been diagnosed by bronchial biopsy of the specimen (Aida et al. 2008). To date, the major issue in bronchial papilloma is that both the clinical presentation and the analysis of the literature show protean symptoms and chest radiographs presentation (Yıldırım et al. 2015; Aida et al. 2008; Paganin et al. 2009). Many patients of bronchial papilloma have symptoms such as cough, dyspnea, wheezing, hemoptysis, or recurrent pneumonia for bronchial obstruction and lobar collapse, and their radiological findings show bronchiectasis or bronchial stenotic change that may be secondary to the recurrent pneumonia (Yıldırım et al. 2015; Paganin et al. 2009). Our patient has productive cough and fever, but this symptom has been considered as the clinical presentation of lung abscess for the abnormal shadow on the patient’s chest radiograph.

Bronchial papilloma is seen more frequently in men and generally appears in the age range of 50–70 years. The treatment methods for bronchial papilloma, such as endoscopic resection or surgical resection, are still controversial for the pathological examination of the whole papilloma can be prevented by laser irradiation while the extent of surgical resection is also a serious consideration, because these benign tumors are mostly located at the main bronchus or orifice of the lobar bronchus (Yıldırım et al. 2015; Harris and Chalhoub 2011; Paganin et al. 2009). In this case of a 48-year old man, for it is complicated with suspicious lung abscess and the patient’s own choice, the lobectomy has been performed instead of the endoscopic intervention (Paganin et al. 2009).

Of particular interest, after surgical resection, the lesion of the right upper lobe in our case is a PSH proved by histopathological examination. As widely known, PSH is a rare benign tumor of the lung, which occurs predominantly in middle aged women (Guerra-Gutiérrez et al. 2007). In most cases, PSH is detected by chance because it is generally asymptomatic as is the case in our patient who is a 48-years-old man with fever and cough, and found by surgical resection for the treatment of another rare benign tumor. Therefore, it is important that the diagnosis of PSH is preoperatively established whenever possible because a limited, but complete, resection of the lesion is the treatment of choice. PSH has the characteristics of a benign pulmonary mass, including a well-defined round or oval mass on radiography, a well-circumscribed lesion with marked contrast enhancement on CT and calcification might be detected in the minority of cases as found in our case (Shin et al. 2014; Zhu et al. 2010). We here report the case of a PSH radiologically presenting as a cystic lesion that has been indicated by Qian et al. (2012), and suggest the existence of a connection between the lesion and bronchi which caused the symptom of productive cough.

Our immunohistochemical results have suggested that the tumor cells stained positively for TTF-1, EMA, CK7, cytokeratin and CD31 that indicate the tumor originates from the epithelium, especially from type-II pneumocytes or Clara cells, but not from endothelium and the mass was decided as a sclerosis haemangioma of lung. As noted above, bronchial papilloma and PSH have been reported respectively in the literature. To the best of our knowledge, we introduce the case of a patient with the diagnosis of bronchial papilloma and PSH.

Although PSH has not been previously associated with bronchial papilloma, cases of PSH co-occurring with myomas, or with thyroid and kidney cysts have been reported in the literature, and these could indicate that PSH is yet another of the malformations that might be present in bronchial papilloma while other reports also have revealed that PSH has been found in association with Cowden syndrome or Lynch syndrome (Guerra-Gutiérrez et al. 2007; Schiergens et al. 2011). However, the pathophysiology of the association between bronchial papilloma and PSH is unclear.

In conclusion, this is a reported case of PSH in a male suffering from bronchial papilloma which is the exceptional concurrence of these two extremely infrequent events. This case highlights the fact that even though some of these lesions may have a low prevalence, they may be included in the differential diagnosis of solitary lung lesions. Nonetheless, the low prevalence of both diseases has meant that it has not been possible to explore the depth of association between them.
